# Evaluation of Nine Commercial Serological Tests for the Diagnosis of Human Hepatic Cyst Echinococcosis and the Differential Diagnosis with Other Focal Liver Lesions: A Diagnostic Accuracy Study

**DOI:** 10.3390/diagnostics11020167

**Published:** 2021-01-25

**Authors:** Francesca Tamarozzi, Silvia Stefania Longoni, Ambra Vola, Monica Degani, Stefano Tais, Eleonora Rizzi, Marco Prato, Salvatore Scarso, Ronaldo Silva, Enrico Brunetti, Zeno Bisoffi, Francesca Perandin

**Affiliations:** 1 Department of Infectious Tropical Diseases and Microbiology, IRCCS Sacro Cuore Don Calabria Hospital, 37024 Verona, Italy; silvia.longoni@sacrocuore.it (S.S.L.); monica.degani@sacrocuore.it (M.D.); stefano.tais@sacrocuore.it (S.T.); eleonora.rizzi@sacrocuore.it (E.R.); marco.prato@sacrocuore.it (M.P.); salvatore.scarso@sacrocuore.it (S.S.); ronaldo.silva@sacrocuore.it (R.S.); zeno.bisoffi@sacrocuore.it (Z.B.); francesca.perandin@sacrocuore.it (F.P.); 2 Department of Medical Sciences and Infectious Diseases, IRCCS San Matteo Hospital Foundation, 27100 Pavia, Italy; ambra.vola@gmail.com (A.V.); enrico.brunetti@unipv.it (E.B.); 3 Department of Clinical, Surgical, Diagnostic and Pediatric Sciences, University of Pavia, 27100 Pavia, Italy; 4 Department of Diagnostics and Public Health, University of Verona, 37129 Verona, Italy

**Keywords:** cystic echinococcosis, *Echinococcus granulosus s.l.*, serology, serodiagnosis, differential diagnosis, diagnostic accuracy, focal liver lesions

## Abstract

The differential diagnosis of hepatic cystic echinococcosis (CE) may be challenging. When imaging is insufficient, serology can be applied, but no consensus diagnostic algorithm exists. We evaluated the performances of nine serological tests commercialized in Europe for the diagnosis of “echinococcosis”. We performed a diagnostic accuracy study using a panel of sera from patients with hepatic CE (*n* = 45 “liquid” content stages, *n* = 25 “solid” content stages) and non-CE focal liver lesions (*n* = 54 with “liquid” content, *n* = 11 with “solid” content). The diagnosis and staging of CE were based on ultrasound (gold standard). Nine commercial seroassays (5 ELISA, 2 WB, 1 Chemiluminescence Immunoassay [CLIA] and 1 Immunochromatographic test [ICT]) were the index tests. Sensitivity (Se) ranged from 43 to 94% and from 31 to 87%, and specificity (Sp) from 68 to 100% and from 94 to 100%, when borderline results were considered positive or negative, respectively. Three seroassays (2 ELISA, 1 WB) were excluded from further analyses due to poor performances. When tests were combined, Sp was 98–100%. The best results were obtained using the WB-LDBIO alone (Se 83%) or as a third test after two non-WB tests (Se 67–86%). A validated WB or two non-WB tests, read with stringent criteria (borderline = negative and considered positive only if concordant positive), possibly confirmed by the WB, appear sensible approaches.

## 1. Introduction

Cystic echinococcosis (CE) is caused by infection with the larval stage of *Echinococcus granulosus sensu lato*. This develops as fluid-filled cysts, most frequently in the liver, after accidental ingestion of parasite eggs [1]. The parasite life cycle develops mostly between domestic dogs and livestock ungulates, and the infection is prevalent worldwide where livestock breeding is practiced [2,3]. The spectrum of clinical manifestations of human CE ranges from being asymptomatic to causing serious, disabling pathology; when present, symptoms are nonspecific [4].

CE cysts pass through different stages, from unilocular, fluid-filled CE1, to more “complex” stages (CE2, CE3a, CE3b), and “solid” inactive stages (CE4, CE5) [5]. Ultrasound is at the basis of the diagnosis and staging of abdominal CE, which are pivotal to guide clinical decision-making [5,6]. Pathognomonic features of CE can be visualized with ultrasound; however, in their absence or when clinicians are not experienced in recognizing them, the diagnosis of CE may be difficult. The wide spectrum of differential diagnoses ranges from biliary cysts to malignancies. Serology is applied to support imaging in doubtful cases; however, the interpretation of serological test results may be challenging. Tests are not standardized, often lack appropriate validation, and their performances vary widely [7]. Furthermore, serology should be applied only after a lesion is visualized on imaging; however, most seroassay performance studies were not designed to fulfill this criterion [8]. False positive reactions may occur, with frequent cross-reactivity in case of infection with *E. multilocularis*, causing alveolar echinococcosis (AE) [7,9,10]. Misdiagnosis of CE and AE [11], and likewise of other etiologies, may have dramatic consequences. Finally, sensitivity is influenced by a number of factors, resulting in false negative results [7,12,13]. Therefore, a negative serology may occur even in the presence of active CE.

Serology results must be interpreted in the light of imaging characteristics and pre-test probabilities of a lesion being CE or another etiology [13,14]. At present, no evidence-based consensus algorithm exists to guide the use of serological tests in the diagnostic process of CE-evocative lesions. In a retrospective study, it has been suggested that the application of a Western blot test, as a single-test approach or as a confirmation after two first-level tests, might have the best performance [14].

Here, we aim to evaluate the performances of nine serological tests commercialized in Europe for the diagnosis of “echinococcosis”, using a panel of well-characterized sera from patients with hepatic CE and with non-CE lesions potentially in differential diagnosis with CE. The work is presented according to STARD (Standards for Reporting Diagnostic accuracy studies) recommendations [15].

## 2. Materials and Methods

### 2.1. Ethics Statement

All patients signed the informed consent for storage and research use of the leftover serum when blood was sampled for routine analyses. Approval was granted by the Ethics Committees of San Matteo Hospital, Pavia (n. 20150004877) and of Verona and Rovigo Provinces (n. 66411 of 25/11/2019), Italy.

### 2.2. Samples Panel and Classification

This is a diagnostic accuracy study performed on stored sera from patients with hepatic CE and nonparasitic focal hepatic lesions, visited between 2012–2019 in the Department of Infectious Diseases, IRCCS San Matteo Hospital Foundation, Pavia, and the Department of Infectious-Tropical Diseases and Microbiology (DITM), IRCCS Sacro Cuore Don Calabria Hospital, Negrar, Italy. Sera were stored at −80 °C and thawed ≤2 times before use. Patients and samples, if available, were selected based on: (1) presence of ≥1 focal liver lesion of nonparasitic (controls) or CE (cases) etiology; (2) available ultrasound (US) images and/or reports defining the non-CE lesion or the staging of the CE cyst(s) according to the WHO-IWGE (WHO Informal Working Group on Echinococcosis) classification [5]; (3) whenever possible, serum collected from patients with CE who were treatment-naïve or whose treatment started <15 days or ended >12 months before serum collection [11]. If multiple hepatic CE cysts were present, the case was classified according to the stage of the cyst known to be most commonly associated with positive serology [13]. Cases were then grouped into having “liquid” active (CE1, CE2, CE3a, CE3b) or “solid” inactive (CE4, CE5) CE. Controls were classified as having “liquid” or “solid” focal liver lesions. Anonymized details of the included sera/patients are available in File S1.

### 2.3. Diagnostic Tests

Sera were analyzed using nine commercially available tests (Table S1): five IgG Enzyme-linked Immunosorbent Assays (ELISA; DRG Instruments, Marburg, Germany; EUROIMMUN, Lubeck, Germany; Vircell, Grenada, Spain; IBL International, Hamburg, Germany; R-Biopharm, Darmstadt, Germany), two Western Blot (WB; LDBIO Diagnostics, Lyon, France; EUROIMMUN, Lubeck, Germany), one Chemiluminescence Immunoassay (CLIA; Vircell, Grenada, Spain), and one Immunochromatographic (ICT) rapid diagnostic test (RDT; Vircell, Grenada, Spain). All tests were donated from the manufacturers, with the exception of DRG ELISA, which is routinely in use in the DITM lab, and of LDBIO WB, which was 50% waived. No manufacturer had any role in sample processing, data analysis or results interpretation. The laboratory operators were blind to the classification of the samples and to the results of other tests/readings.

For the ELISA assays (Table S1), sera were tested in single and Optical Densities read using the ELx800 ELISA reader (BioTek Instruments, Winooski, VT, USA). The ECHINOCOCCUS Western blot IgG (LDBIO Diagnostics), was performed using the DynaBlot Plus (DYNEX TECHNOLOGIES, Prague, Czech Republic) instrument. One experienced laboratory operator read and interpreted the bands (Table S1). In case of doubt, the strip was read by a second operator and, in case of discordant interpretation, by a third operator, as per routine procedure at DITM laboratory. The Anti-Echinococcus EUROLINE WB IgG (EUROIMMUN) was read visually and using the EUROLineScan dedicated software. For visual reading, due to the complexity of the procedure, each strip was read by two experienced laboratory operators, and, in case of interpretation leading to discordant results, by a third operator (Table S1). The HYDATIDOSIS VIRCLIA^®^ IgG MONOTEST (Vircell) was performed using the VIRCLIA^®^ Powerful Automation System (Thunderbolt^®^) and results processed by the dedicated software. The VIRapid HYDATIDOSIS (Vircell) assay was read by two experienced operators and by a third operator in case of pos/neg discordant reading (Supplementary Materials Table S1).

### 2.4. Statistical Analysis

Patients/samples formed a convenience series limited by availability of 135 stored sera. The diagnosis and staging of CE were based on the US findings, before serology results were available. Tests sensitivities and specificities were calculated considering US as the gold standard; seroassays were index tests. Pre-test and post-test probabilities were estimated by simulating the application of the index assays in areas of low (0.5%) and mid-high (5%) prevalence of CE. We considered that 50% of CE cysts would be active and 50% inactive [16]. We also considered 2.5% the prevalence of biliary cysts and 5% the prevalence of hemangioma (arguably the most common focal “liquid” and “solid” liver lesion, respectively) in the population [16,17,18,19,20,21,22]. Diagnostic accuracy analyses were performed interpreting borderline/gray-zone/indecisive (thereafter “borderline”) results as either positive or negative. When tests were combined, the final diagnosis (CE/non-CE) was interpreted either with a “concordant-positive” approach (i.e., diagnosis of CE in case of 2 concordant positive tests) or with a “one-positive-only” approach (i.e., diagnosis of CE in case of just 1 positive test). Parameters are reported with 95% confidence interval (CI) and statistical significance level fixed at 0.05. The SAS software version 9.4 (Cary, NC, USA) was used for the statistical analysis.

## 3. Results

### Serum Samples Cohort

**Figure 1:**
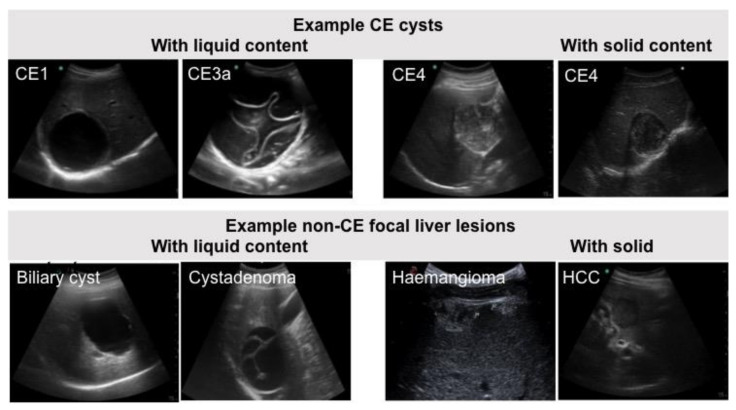
Example ultrasound images of focal liver lesions in differential diagnosis with CE. Top panel: CE cysts in active (CE1) and transitional (CE3a) stages, having a liquid content, and inactive (CE4) stage with solid appearance. Lower panel: focal liver lesions of other etiology with liquid (biliary cyst and cystadenoma) and solid (atypical haemangioma) appearance. HCC: hepatocellular carcinoma.

Patients and sera characteristics are detailed in File S1. A total of 135 sera were analyzed, 65 from patients with non-CE focal hepatic lesions (controls) and 70 from patients with hepatic CE (cases). Forty-five cases were classified as having “liquid” active CE (CE1 *n* = 9, CE2 *n* = 10, CE3a *n* = 12, CE3b *n* = 14) and 25 having “solid” inactive CE (CE4 *n* = 16, CE5 *n* = 9) (Figure 1). Fifty-four (83.1%) control sera were from individuals with “liquid” and 11 (16.9%) with “solid” focal hepatic lesions (Figure 1). No sera from patients with AE were available. Due to limited availability of serum, only 126 sera could be tested with the ICT assay, 62 from cases and 64 from controls.

## 4. Evaluation of the Diagnostic Accuracy of Assays

### 4.1. Single Tests

**Figure 2:**
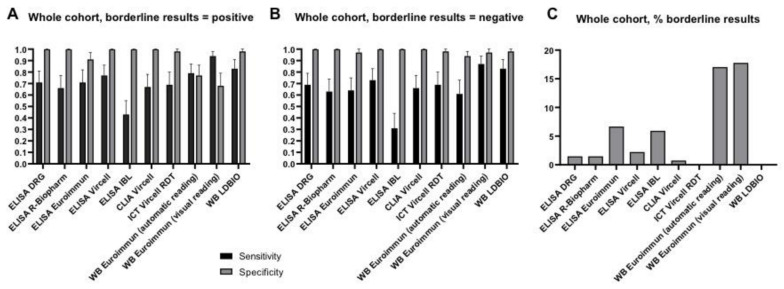
Diagnostic performance of the single index tests, on the whole sera cohort. Sensitivity and specificity of assays when borderline results were considered as positive (**A**) and negative (**B**). Percentage of borderline results (**C**). Error bars represent 95% CI.

The performances of each test are shown in Figure 2 and Figure S1, and detailed in Files S2 and S3. Sensitivity ranged from 43 to 94% and from 31 to 87%, and specificity from 68 to 100% and from 94 to 100%, when borderline results were considered as positive or negative, respectively. Sensitivities were higher in the “active CE-liquid lesions” compared to the “inactive CE-solid lesions” subgroup (Figure S2).

The IBL ELISA had the lowest sensitivity while specificity was lowest for the Euroimmun WB and the Euroimmun ELISA. The highest rate of doubtful results was observed for the Euroimmun WB (>17%), followed by the Euroimmun ELISA (6.67%) and the IBL ELISA (5.93%) (Figure 2). The rate of doubtful results was comparable in the “active CE-liquid lesions” and in the “inactive CE-solid lesions” groups (Figure 2). The Euroimmun WB had also the highest rate (2.33–3.28%) of *E. multilocularis* species misidentification in sera from CE cases, compared with 1.72% of LDBIO WB. Furthermore, with the Euroimmun WB automatic reading, all (*n* = 4) false positive results were identified as *E. multilocularis* (File S2). Overall, the visual reading of the Euroimmun WB was very demanding, with about half of tests having bands read differently between operators (File S2). In the light of these results, Euroimmun WB, Euroimmun ELISA, and IBL ELISA were not further evaluated.

### 4.2. Combination of Two Tests

Due to the risk of false positive results, especially in patients from areas of high CE prevalence [23,24], the combination of at least two concordant positive tests to confirm the diagnosis of CE seems a sensible strategy, as also applied for other infections [25,26].

**Figure 3:**
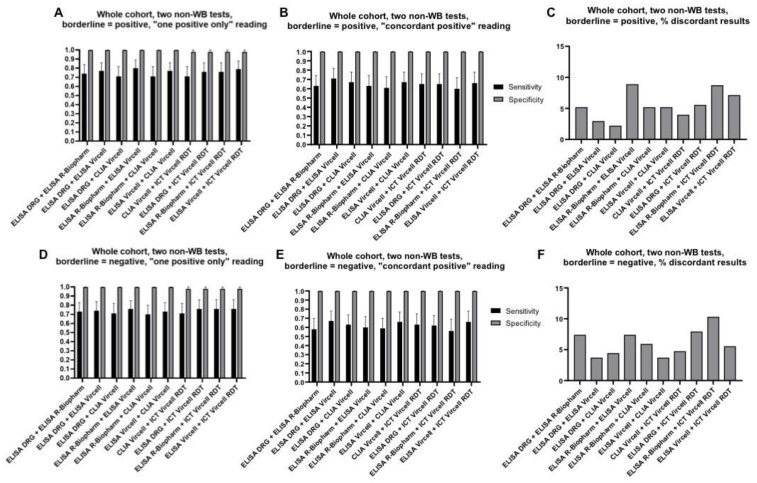
Diagnostic performance of the two non-WB index tests, on the whole sera cohort. Sensitivity and specificity of assays when borderline results were considered as positive and final result interpreted using the “one positive only” approach (**A**) or the “concordant positive” approach (**B**); and when borderline results were considered as negative and final result interpreted using the “one positive only” approach (**D**) or the “concordant positive” approach (**E**). Percentage of discordant results when borderline results were considered as positive (**C**) or negative (**F**). Error bars represent 95% CI. Of note, CLIA Vircell and ELISA Vircell contain the same antigenic preparation, therefore its combined use should be avoided; data relative to this combination are presented for completeness.

WB is expensive, may not be widely available, and requires trained personnel for its interpretation. Therefore, firstly we evaluated the diagnostic performance of the combination of two tests different from WB. Results are shown in Figure 3 and detailed in File S3. Sensitivity ranged from 58 to 67% (borderline = negative, “concordant-positive” approach) to 71–80% (borderline = positive, “one-positive-only” approach). Specificity was very high (98–100%) for all combinations and independently of the interpretation approach.

**Figure 4:**
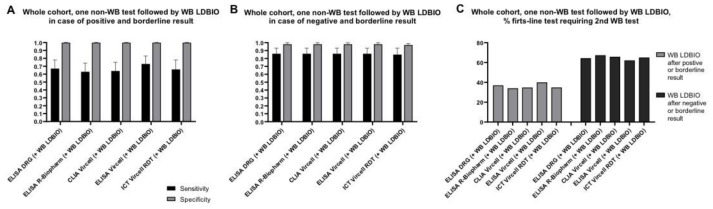
Diagnostic performance of the combination of each non-WB assay followed by LDBIO WB, on the whole sera cohort. Sensitivity and specificity of assays when borderline results were considered as positive (**A**) and negative (**B**). Percentage of first-level non-WB tests requiring a second WB test (**C**). Error bars represent 95% CI. Of note, CLIA Vircell and ELISA Vircell contain the same antigenic preparation, therefore its combined use should be avoided; data relative to this combination are presented for completeness.

WB is commonly used to confirm the positive result of a first-level test. We therefore explored the diagnostic accuracy of the combination of each non-WB assay followed by the LDBIO WB in case of positive or borderline result. Results are shown in Figure 4 and detailed in File S3. Sensitivity ranged from 63 to 73% with mean 36% first-level tests requiring an additional analysis by LDBIO WB. We then explored whether sensitivity could be improved by applying the WB on negative and borderline first-level tests results. Sensitivity improved to 85–86%, but mean 65% of first-level tests would require an additional analysis by WB (Figure 4 and File S3).

### 4.3. Combination of Three Tests

**Figure 5:**
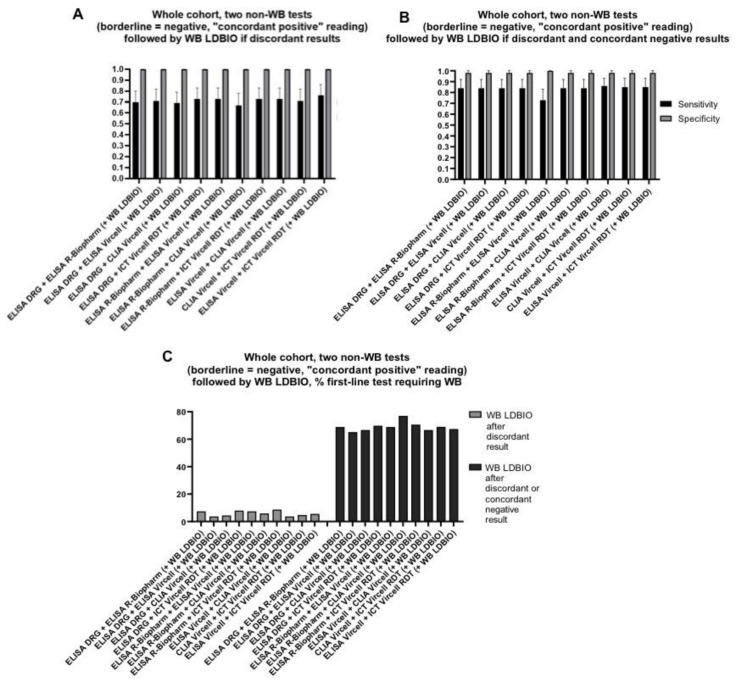
Diagnostic performance of the combination of two first-line non-WB assays (borderline results considered as negative and “concordant positive” interpretation approach applied) followed by LDBIO WB, on the whole sera cohort. Sensitivity and specificity of assays when WB was applied only following discordant first-level tests results (**A**) and following both discordant and concordant negative results (**B**). Percentage of first-level non-WB tests combinations requiring a following WB test (**C**). Error bars represent 95% CI. Of note, CLIA Vircell and ELISA Vircell contain the same antigenic preparation, therefore its combined use should be avoided; data relative to this combination are presented for completeness.

It has been suggested that the application of a confirmative WB test after two first-level tests might have the best performance for the diagnosis of hepatic CE [14]. We therefore evaluated the performance of this approach. Specificity was very high (98–100%) for all combinations and interpretations. In case WB was applied only if the first 2 tests had discordant results, sensitivity ranged from 69 to 76% with mean 6% first-line tests requiring a WB analysis. In case WB was applied on both discordant and concordant negative results of the first two tests, sensitivity ranged from 73 to 86% with mean 69% first-line tests requiring further WB analysis. Results are shown in Figure 5 and detailed in File S3.

## 5. Simulation of Post-Test Probabilities

Pre-test and post-test probabilities are more readily interpretable concepts in clinical practice than sensitivity and specificity. We therefore explored the probabilities of a positive and negative serology result to correspond to CE infection, in patients with “liquid”- or “solid”-appearing focal liver lesions. Results are shown in Figure S3. The probability of a patient with evocative focal liver lesions of having CE in case of positive serology was high (83–100%). The probability of a patient having CE despite a negative serology was low in the presence of active CE (1–3%) but not negligible in the case of inactive CE (10–23%).

## 6. Discussion

The diagnosis of CE may be challenging due to the different morphology of CE cysts, their wide spectrum of differential diagnoses, and the often little familiarity of physicians with this infection. Serology is applied when the diagnosis cannot be based on imaging alone. The current WHO-IWGE Expert Consensus [5] recommends the application of “a high-sensitivity serological test, confirmed by a separate high-specificity serological test”; however, no consensus algorithm is available to guide the application and interpretation of serology. Based on a retrospective analysis of serology results, it was suggested that WB, alone or to confirm the result of two first-line tests, might have the best performance [14]. Here, we examined the diagnostic performances of nine serological tests commercialized in Europe for the diagnosis of “echinococcosis”, and their combination, using a panel of well-characterized, clinically appropriate sera.

We did not pre-set performance thresholds to define an assay as acceptable; however, we excluded three tests from the combination analysis due to their unsatisfactory performances. For all other tests, specificity was very high. This is not surprising because control sera were from selected patients with hepatic focal lesions, coming from areas not endemic for CE, and did not include patients with AE. Therefore, the results of our study may apply to the clinical settings in low/nonendemic areas for CE, but should be translated with caution to high-CE or CE/AE co-endemic areas.

Several papers reported the diagnostic performance of some of the commercial seroassays evaluated in this work [8,10,13,14,27,28,29,30]. As diagnostic performances substantially depend on the characteristic of CE cysts and controls forming the examined sample cohort [13], it is difficult to directly compare the results of studies, even more so when cyst stages and other relevant factors are not described and the aims of the studies differ. However, diagnostic accuracy parameters found in this study were, overall, within the reported ranges for those tests previously evaluated in the published literature [8,10,13,14,27,28,29,30].

In accordance with previous results of a study with comparable design [14], the LDBIO WB assay resulted the best single test to apply in the presence of suggestive focal liver lesions (Se 83%, 95% CI 72–91%; Sp 98%, 95% CI 91–100%). Of note, the visible band identifying the only false positive result of this test was faint and irregular; therefore, the specificity of the assay was probably underestimated in this study. When considering other assays and combinations, higher sensitivities were, logically, achieved when borderline results were considered as positive and using the “one-positive-only” approach. However, this approach should probably not be recommended in all settings, especially in areas of intense *E. granulosus* circulation and co-endemicity with potentially cross-reactive parasitoses, where false positive results are expected to be much more frequent. Since a diagnosis of CE cannot be excluded by a negative serology and prompts further investigations, a false negative result would be a more “cautionary” result. We therefore privileged the interpretation of borderline results as negative and of test combinations using the “concordant-positive” approach. When applying these stringent criteria, the application of two first-level tests followed by WB in case of discordant results (Se 67–73%, 95% CI 55–86%; Sp 100%, 95% CI 94–100%), or in case of discordant and concordant negative results (Se 73–86%, 95% CI 61–93%; Sp 98–100%, 95% CI 92–100%), had the best performances.

Deserving a final mention is the accuracy of *Echinococcus* species identification based on WB banding pattern. It is well known that WB can discriminate CE and AE only in a percentage of cases [10]; furthermore, our results show that a variable proportion of sera from patients with CE and from uninfected patients can be identified as AE based on WB banding pattern. This is of great concern because CE and AE (and other causes of focal liver lesions) have very different clinical management and prognoses. Therefore, a very cautious interpretation of WB banding pattern for *Echinococcus* species identification is absolutely needed, which in no way can constitute the sole basis of species identification.

This study had several limitations. Sera from patients with AE were unavailable; furthermore, controls did not come from the same areas of patients with CE. This may have been responsible for the very high specificity of all tests, and highlights the need to perform a similar study in CE-endemic and CE/AE co-endemic areas. We could not exclude pulmonary CE in control patients. This possible source of false-positive results, however, seems of marginal concern, because very few false-positives were obtained and because anyway lung CE is associated with a low rate of seropositivity [31]. Finally, half of sera from patients with CE came from patients who received albendazole treatment, one third of which recently (File S1), that may have increased tests sensitivities [13]. On the other hand, the strength of this study is the use of a relatively large sample size of sera, from patients with well-characterized lesions, and selected using stringent criteria, to minimize the possible influence of previous treatment on serology results. This allowed evaluating assays performance in the conditions most similar to a first diagnosis of the focal liver lesion.

## 7. Conclusions

To conclude, a WB appears the best test to apply when a one-test approach is chosen. The performance of two first-level tests, read with stringent criteria and possibly confirmed by a validated WB, appears similarly sensible. However, careful verification of assays performance, including of WB assays, using a panel of well-characterized sera appropriate to the clinical-epidemiological setting, should be carried out before introducing any test in clinical practice. Similar studies should be carried out in CE-endemic and CE/AE co-endemic areas and using a prospective, diagnostic-benefit design.

## Data Availability

All anonymized data are available in the supplementary material.

## Supplementary Materials

The following are available online at https://www.mdpi.com/2075-4418/11/2/167/s1, Figure S1: Heat map representation of results of all index tests in individual sera, Figure S2: Diagnostic performance of single index tests by patients’ subgroups, Figure S3: Probability of a positive (red arrow) and negative (blue arrow) test to indicate a diagnosis of active or inactive CE when representative combinations of assays are applied in low and mid-high prevalence of CE, Table S1: Description of the evaluated serological tests. File S1: Sera and patients’ details, File S2: Serology assays results, File S3: Sensitivity, specificity, and post-test probability analyses details.

## References

[1] Casulli A., Siles-Lucas M., Tamarozzi F. (2019). Echinococcus granulosus sensu lato. Trends Parasitol..

[2] Deplazes P., Rinaldi L., Alvarez Rojas C.A., Torgerson P.R., Harandi M.F., Romig T., Antolova D., Schurer J.M., Lahman S., Cringoli G. (2017). Global distribution of alveolar and cystic echinococcosis. Adv. Parasitol..

[3] Craig P.S., Hegglin D., Lightowlers M.W., Torgerson P.R., Wang Q. (2017). Echinococcosis: Control and prevention. Adv. Parasitol..

[4] Kern P., Menezes da Silva A., Akhan O., Mullhaupt B., Vizcaychipi K.A., Budke C., Vuitton D.A. (2017). The echinococcoses: Diagnosis, clinical management and burden of disease. Adv. Parasitol..

[5] Brunetti E., Kern P., Vuitton D.A., Writing Panel for the WHO-IWGE (2010). Expert consensus for the diagnosis and treatment of cystic and alveolar echinococcosis in humans. Acta Trop..

[6] Stojković M., Weber T.F., Junghanss T. (2018). Clinical management of cystic echinococcosis: State of the art and perspectives. Curr. Opin. Infect. Dis..

[7] Siles-Lucas M., Casulli A., Conraths F.J., Muller N. (2017). Laboratory diagnosis of *Echinococcus* spp. in human patients and infected animals. Adv. Parasitol..

[8] Tamarozzi F., Mariconti M., Covini I., Brunetti E. (2017). Rapid diagnostic tests for the serodiagnosis of human cystic echinococcosis. Bull. Soc. Pathol. Exot..

[9] de la Rue M.L., Yamano K., Almeida C.E., Iesbich M.P., Fernandes C.D., Goto A., Kouguchi H., Takahashi K. (2010). Serological reactivity of patients with *Echinococcus* infections (*E. granulosus, E. vogeli*, and *E. multilocularis*) against three antigen B subunits. Parasitol. Res..

[10] Liance M., Janin V., Bresson-Hadni S., Vuitton D.A., Houin R., Piarroux R. (2000). Immunodiagnosis of *Echinococcus* infections: Confirmatory testing and species differentiation by a new commercial Western Blot. J. Clin. Microbiol..

[11] Stojkovic M., Mickan C., Weber T.F., Junghanss T. (2015). Pitfalls in diagnosis and treatment of alveolar echinococcosis: A sentinel case series. BMJ Open Gastroenterol..

[12] Hernandez-Gonzalez A., Muro A., Barrera I., Ramos G., Orduna A., Siles-Lucas M. (2008). Usefulness of four different *Echinococcus granulosus* recombinant antigens for serodiagnosis of unilocular hydatid disease (UHD) and postsurgical follow-up of patients treated for UHD. Clin. Vaccine Immunol..

[13] Lissandrin R., Tamarozzi F., Piccoli L., Tinelli C., De Silvestri A., Mariconti M., Meroni V., Genco F., Brunetti E. (2016). Factors influencing the serological response in hepatic *Echinococcus granulosus* infection. Am. J. Trop. Med. Hyg..

[14] Vola A., Manciulli T., De Silvestri A., Lissandrin R., Mariconti M., Siles-Lucas M., Brunetti E., Tamarozzi F. (2019). Diagnostic performances of commercial ELISA, Indirect Hemagglutination, and Western Blot in differentiation of hepatic echinococcal and non-echinococcal lesions: A retrospective analysis of data from a single referral centre. Am. J. Trop. Med. Hyg..

[15] Equator Network. https://www.equator-network.org/reporting-guidelines/stard/.

[16] Chebli H., El Laamrani Idrissi A., Benazzouz M., Lmimouni B.E., Nhammi H., Elabandouni M., Youbi M., Afifi R., Tahiri S., El Feydi A.E. (2017). Human cystic echinococcosis in Morocco: Ultrasound screening in the Mid Atlas through an Italian-Moroccan partnership. PLoS Negl. Trop. Dis..

[17] Bajenaru N., Balaban V., Savulescu F., Campeanu I., Patrascu T. (2015). Hepatic hemangioma-review. J. Med. Life.

[18] Kaltenbach T.E., Engler P., Kratzer W., Oeztuerk S., Seufferlein T., Haenle M.M., Graeter T. (2016). Prevalence of benign focal liver lesions: Ultrasound investigation of 45,319 hospital patients. Abdom. Radiol..

[19] Lantinga M.A., Gevers T.J., Drenth J.P. (2013). Evaluation of hepatic cystic lesions. World J. Gastroenterol..

[20] Leon M., Chavez L., Surani S. (2020). Hepatic hemangioma: What internists need to know. World J. Gastroenterol..

[21] Mocchegiani F., Vincenzi P., Coletta M., Agostini A., Marzioni M., Baroni G.S., Giovagnoli A., Guerrieri M., Marmorale C., Risaliti A. (2016). Prevalence and clinical outcome of hepatic haemangioma with specific reference to the risk of rupture: A large retrospective cross-sectional study. Dig. Liver Dis..

[22] Rawla P., Sunkara T., Muralidharan P., Raj J.P. (2019). An updated review of cystic hepatic lesions. Clin. Exp. Hepatol..

[23] Moro P.L., Bonifacio N., Gilman R.H., Lopera L., Silva B., Takumoto R., Verastegui M., Cabrera L. (1999). Field diagnosis of *Echinococcus granulosus* infection among intermediate and definitive hosts in an endemic focus of human cystic echinococcosis. Trans. R. Soc. Trop. Med. Hyg..

[24] Shambesh M.A., Craig P.S., Macpherson C.N., Rogan M.T., Gusbi A.M., Echtuish E.F. (1999). An extensive ultrasound and serologic study to investigate the prevalence of human cystic echinococcosis in northern Libya. Am. J. Trop. Med. Hyg..

[25] Eldin C., Raffetin A., Bouiller K., Hansmann Y., Roblot F., Raoult D., Parola P. (2019). Review of European and American guidelines for the diagnosis of Lyme borreliosis. Med. Mal. Infect..

[26] Lapa J.S., Saraiva R.M., Hasslocher-Moreno A.M., Georg I., Souza A.S., Xavier S.S., do Brasil P.E.A.A. (2012). Dealing with initial inconclusive serological results for chronic Chagas disease in clinical practice. Eur. J. Clin. Microbiol. Infect. Dis..

[27] Baraquin A., Zait H., Grenouillet F.E., Moreau E., Hamrioui B., Grenouillet F. (2017). Large-scale evaluation of a rapid diagnostic test for human cystic echinococcosis. Diagn. Microbiol. Infect. Dis..

[28] Kalantari E., Bandehpour M., Pazoki R., Taghipoor-Lailabadi N., Khazan H., Mosaffa N., Nazaripouya M.R., Kazemi B. (2010). Application of recombinant Echinococcus granulosus antigen B to ELISA kits for diagnosing hydatidosis. Parasitol. Res..

[29] Mahajan S., Thapar S., Khillan V., Gupta P., Rastogi A., Gupta E. (2020). Comparative evaluation of *Echinococcus* serology with cytology for the diagnosis of hepatic hydatid disease. J. Lab. Physicians.

[30] Tamer G.S., Dündar D., Uzuner H., Baydemir C. (2015). Evaluation of immunochromatographic test for the detection of antibodies against *Echinococcosis granulosus*. Med. Sci. Monit..

[31] Gavidia C.M., Gonzalez A.E., Zhang W., McManus D.P., Lopera L., Ninaquispe B., Garcia H.H., Rodríguez S., Verastegui M., Calderon C. (2008). Diagnosis of cystic echinococcosis, central Peruvian Highlands. Emerg. Infect. Dis..

