# Supplementary material for: Evaluation of Nine Commercial Serological Tests for the Diagnosis of Human Hepatic Cyst Echinococcosis and the Differential Diagnosis with Other Focal Liver Lesions: A Diagnostic Accuracy Study
Source: Diagnostics (Basel). 2021 Jan 25;11(2):167. doi: 10.3390/diagnostics11020167 (PMC7911993; doi:10.3390/diagnostics11020167)
Supplement: Supplementary file 1 [file diagnostics-11-00167-s001.zip › diagnostics-1084516-supli-8.pdf]

Commercial name	Manufacturer	Test format*	Result reading	Antigenic preparation
Echinococcus IgG ELISA	DRG Instruments GmbH, Marburg, Germany	ELISA	Qualitative, based on DRG Units calculated on the basis of the absorbance value of the sample and the mean absorbance values of two kit's controls.<br>ELISA Index: Negative <0.9; Borderline 0.9≤x<1.1; Positive ≥1.1 **	*E. granulosus* antigen not further specified
Anti-Echinococcus ELISA (IgG)	EUROIMMUN, Lubeck, Germany	ELISA	Qualitative, based on the ratio between the absorbance value of the sample and the absorbance value of a kit's calibrator.<br>ELISA Index: Negative <0.8; Borderline 0.8≤x<1.1; Positive ≥ 1.1	*E. multilocularis* purified antigens not further specified
HYDATIDOSIS ELISA IgG	Vircell, Granada, Spain	ELISA	Qualitative, based on Antibody Index calculated on the basis of the ratio between the absorbance value of the sample and the mean absorbance value of a kit's control.<br>ELISA Index: Negative <0.9; Borderline 0.9≤x<1.1; Positive ≥1.1 **	*E. granulosus* cyst fluid from sheep, purified by HPLC
EchinococcusIgG ELISA	IBL International GmbH, Hamburg, Germany	ELISA	Qualitative and qualitative, based on Cut-Off Index calculated in the basis of the ration between the absorbance value of the sample and the absorbance value of a kit's control.<br>ELISA Index: Negative <0.8; Borderline 0.8≤x<1.2; Positive ≥1.2	"Specific antigen", not further specified
RIDASCREEN EchinococcusIgG	R-Biopharm AG, Darmstadt, Germany	ELISA	Qualitative, based on Antibody Index calculated on the basis of the ratio between the absorbance value of the sample and an index obtained from the mean absorbance of two kit's controls and a constant.<br>ELISA Index: Negative <0.9; Borderline 0.9≤x<1.1; Positive ≥1.1	Antigens from *E. granulosus* cysts, not further specified
VIRAPID HYDATIDOSIS	Vircell, Granada, Spain	ICT	Qualitative (positive-negative) based on the comparison of test band intensity with a reference intensity card°	Antigen 5/B of *E. granulosus* purified and enriched by HPLC
ECHINOCOCCUS Western blot IgG	LDBIO Diagnostics, Lyon, France	WB	Qualitative (*Echinococcus spp.*, *E. granulosus* OR *E. multilocularis*; negative) based on the reading of the combination of bands:<br>*Echinococcus spp* = 26-28 kDa or 7kDa and 26-28 kDa<br>*E. granulosus* = 7 kDa or 7 kDa and 17 kDa +/- 26-28 kDa<br>*E. multilocularis* = 26-28 kDa and/or 16 KDa and/or 18 kDa	Antigens of *E. multilocularis* larvae, not further specified
Anti-Echinococcus EUROLINE WB (IgG)	EUROIMMUN, Lubeck, Germany	WB	Qualitative (positive, negative, borderline; *E. granulosus*, *E. multilocularis*, not differentiated)<br>• VISUAL READING: Comparison of the test trip bands with a reference strip for each lot:<br>*Echinococcus spp* = 7 kDa and/or 16/18 kDa and/or 21 kDa<br>*E. granulosus* = at least 1 *Echinococcus spp* band + EgAgB narrow band<br>*E. multilocularis* = at least 1 Echinococcus spp band + Em18 and/or Em95 narrow band<br>BORDERLINE: EgAgB or faint *Echinococcus spp* band<br>• EUROLineScan software applied to a Canon scanner (CanoScan Lide 400) using the reference strip for each lot.	*E. granulosus* antigenic extract (not further specified) and synthetic antigens Em18, Em95, and EgAgB
HYDATIDOSIS VIRCLIA® IgG MONOTEST	Vircell, Granada, Spain	CLIA^	Qualitative (positive, negative, equivocal) based on Antibody Index calculated based on the ratio between the Relative	*E. granulosus* cyst fluid from sheep purified by HPLC

	Chemoluminescent Units of the sample and the Relative Chemoluminescent Units of the kit's calibrator.

Supplementary Table S2. Description of the evaluated serological tests. * ELISA= Enzyme-linked Immunosorbent Assay; ICT=Immunochromatographic test; WB=Western Blot; CLIA=Chemiluminescent Immunoassay. ** Normalized by avoiding x10 multiplication factor in the ELISA Index calculation. ^ For sample volume availability constraints, in this study sera were analyzed in single using 60-150 µl sample volume under the supervision of the Italian VIRCLIA® specialist (Alifax, Polverara, Padua, Italy). ° For the purpose of this study, any clearly visible band was read as positive even if subjectively perceived as fainter than "0.5" intensity band.
