# Supplementary figures and images for: Evaluation of Nine Commercial Serological Tests for the Diagnosis of Human Hepatic Cyst Echinococcosis and the Differential Diagnosis with Other Focal Liver Lesions: A Diagnostic Accuracy Study
Source: Diagnostics (Basel). 2021 Jan 25;11(2):167. doi: 10.3390/diagnostics11020167 (PMC7911993; doi:10.3390/diagnostics11020167)

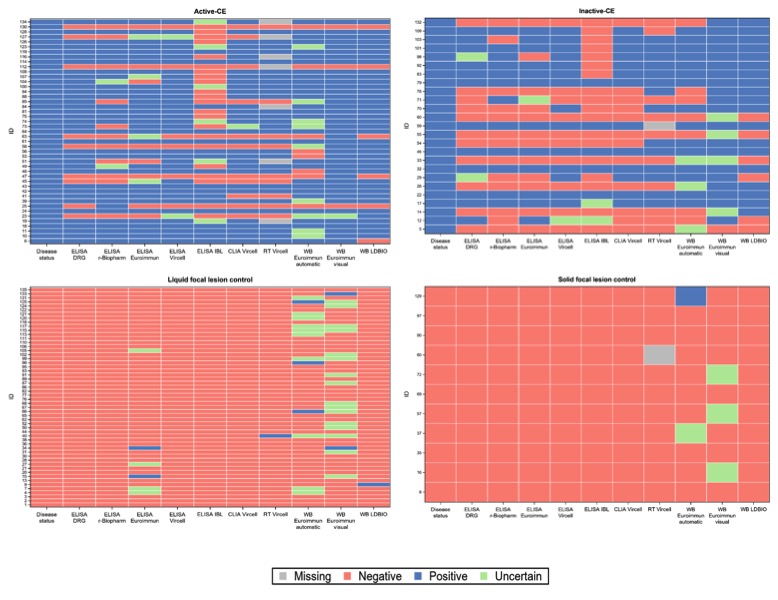

Supplement: Supplementary file 1 [file diagnostics-11-00167-s001.zip › Figure S1.jpg]

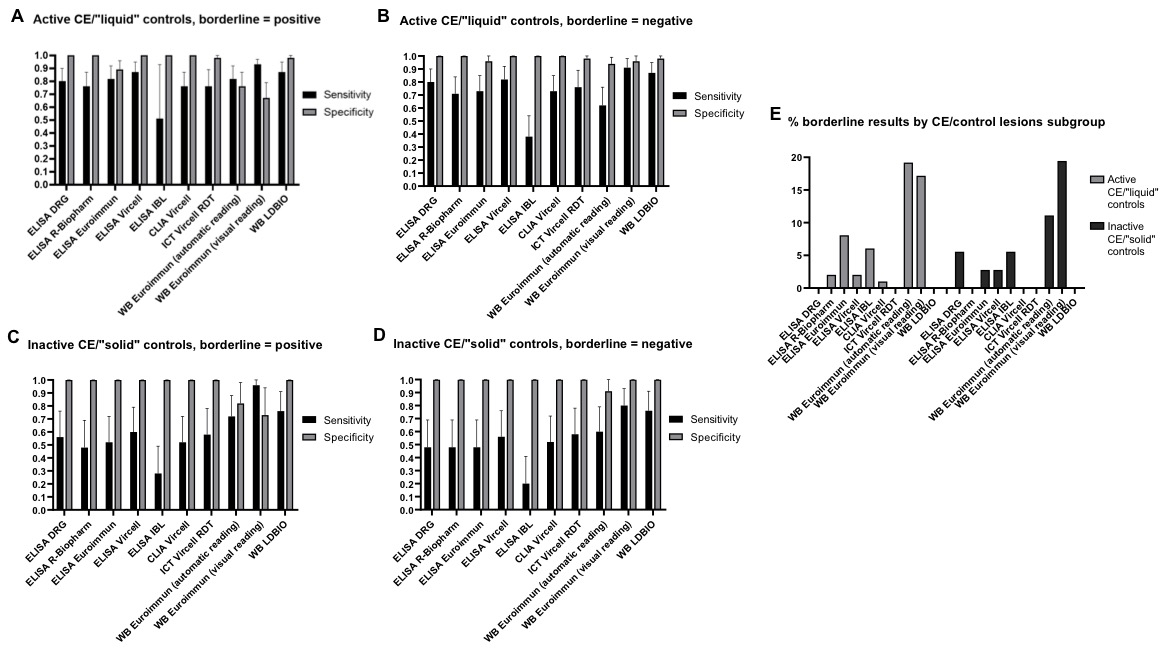

Supplement: Supplementary file 1 [file diagnostics-11-00167-s001.zip › Figure S2.jpg]

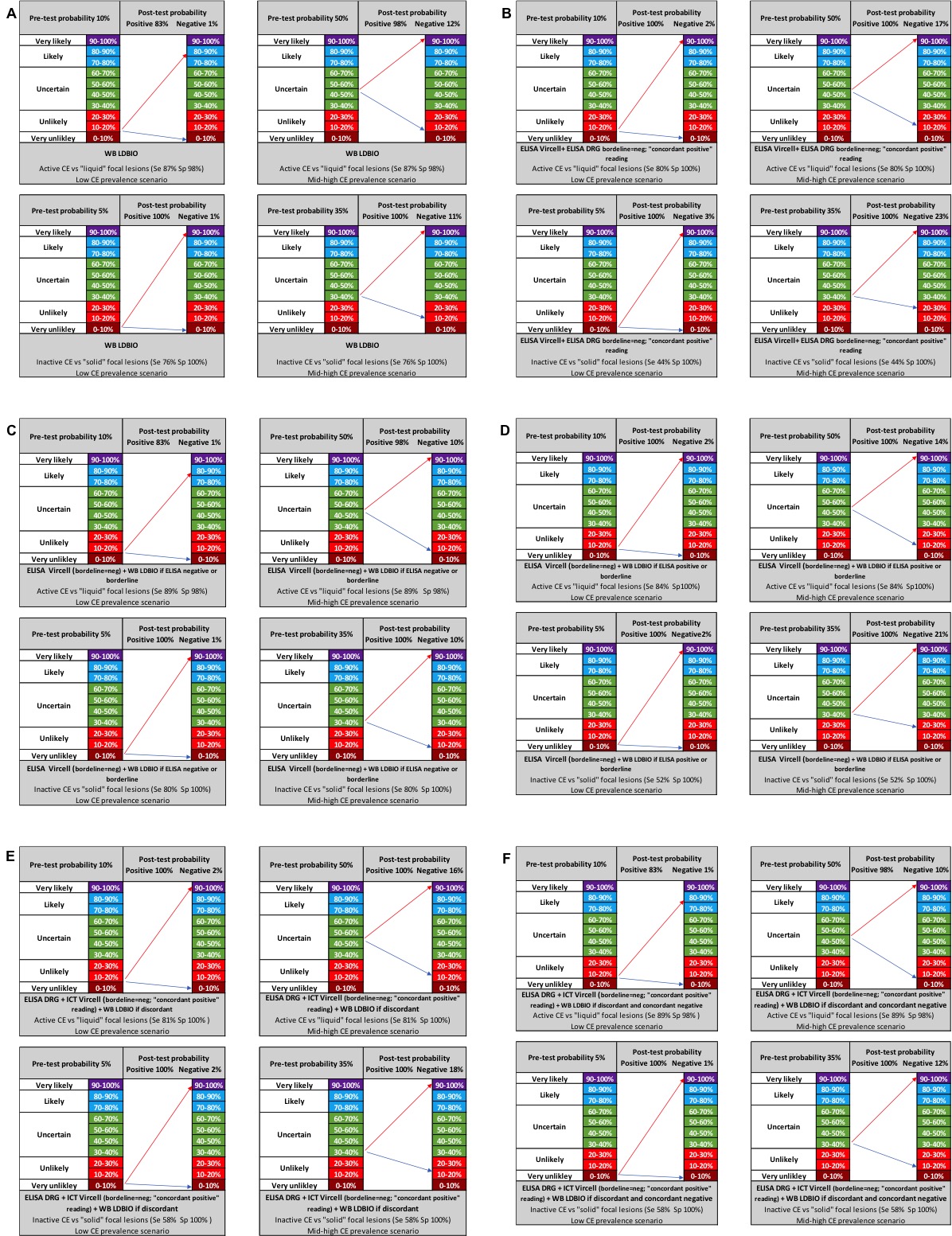

Supplement: Supplementary file 1 [file diagnostics-11-00167-s001.zip › Figure S3.jpg]
